# Estimation of probabilities in favour of pathogenicity for missense substitutions for use in clinical evaluation of mismatch repair gene variants

**DOI:** 10.1186/1897-4287-10-S2-A31

**Published:** 2012-04-12

**Authors:** B Thompson, D Goldgar, C Paterson, M Clendenning, R Walters, S Arnold, M Parsons, M Walsh, J Hopper, M Jenkins, M Greenblatt, D Buchanan, J Young, S Tavtigian, A Spurdle

**Affiliations:** 1 Queensland Institute of Medical Research, Brisbane, Australia; 2 Department of Dermatology, University of Utah, Salt Lake City, USA; 3 Huntsman Cancer Institute, University of Utah, Salt Lake City, USA; 4 School of Medicine, University of Queensland, Brisbane, Australia; 5 Centre for Genetic Epidemiology, University of Melbourne, Melbourne, Australia; 6 Department of Medicine, University of Vermont, Burlington, USA

## 

A considerable proportion of Lynch syndrome families present with mismatch repair (MMR) gene sequence variants of uncertain clinical significance, which constitute a challenge in both the research and clinical settings. Such unclassified variants (UVs) include rare nucleotide changes predicted to cause missense substitutions, small in-frame deletions, or possible alterations in splicing.

We are developing a MMR multifactorial likelihood model to provide a quantitative measure of MMR variant pathogenicity. Bayes analysis of families to measure variant causality by segregation methods is established. Likelihood ratios for microsatellite instability and somatic *BRAF* tumour status have also recently been incorporated into the multifactorial model. We are currently estimating the prior probability of pathogenicity for MMR missense substitutions based on the evolutionary conservation and physicochemical properties of amino acid alterations. To this end, we have built and curated multiple-species sequence alignments of the four MMR proteins. In parallel, we identified 143 apparent missense substitutions, excluded 12 with evidence of causing a splice defect, and then used a combination of quantitative and qualitative criteria to classify 73 as either pathogenic, likely pathogenic, likely not pathogenic, or not pathogenic, based on the IARC five-class system. Six different missense substitution analysis tools (Align-GVGD, MAPP, MutPred, PolyPhen-2.1, SIFT, and Xvir) were used to score the missense substitutions. The bioinformatic outputs are being calibrated by regression against the classifications of the 73 missense substitutions to estimate a prior probability of pathogenicity for MMR missense substitutions.

In summary, we have developed a MMR classification model including tumour characteristics, segregation analysis, and *in silico* prior probability of pathogenicity for missense substitutions. This multifactorial model will be applied to the analysis of 34 UVs from Australasian families, for which we have already completed assessment of tumour pathology, variant association with disease in families, and bioinformatic and *in vitro* splicing analysis. Results from this analysis will alter the management of these and other MMR variant-carrying families.

